# Mining the plasma-proteome associated genes in patients with gastro-esophageal cancers for biomarker discovery

**DOI:** 10.1038/s41598-021-87037-w

**Published:** 2021-04-07

**Authors:** Frederick S. Vizeacoumar, Hongyu Guo, Lynn Dwernychuk, Adnan Zaidi, Andrew Freywald, Fang-Xiang Wu, Franco J. Vizeacoumar, Shahid Ahmed

**Affiliations:** 1 Department of Pathology and Laboratory Medicine, College of Medicine, University of Saskatchewan, Saskatoon, Canada; 2 Division of Biomedical Engineering, University of Saskatchewan, Saskatoon, Canada; 3 Cancer Research, Saskatchewan Cancer Agency, Saskatoon, Canada; 4 Division of Oncology, University of Saskatchewan, Saskatoon, Canada; 5 Department of Mechanical Engineering, University of Saskatchewan, Saskatoon, Canada; 6 Department of Computer Science, University of Saskatchewan, Saskatoon, Canada; 7 Saskatoon Cancer Center, University of Saskatchewan, 20 Campus Drive, Saskatoon, SK S7N4H4 Canada; 8 Saskatoon Cancer Center, University of Saskatchewan, 107 Wiggins Road, Saskatoon, SK S7N5E5 Canada

**Keywords:** Cancer, Cancer genomics

## Abstract

Gastro-esophageal (GE) cancers are one of the major causes of cancer-related death in the world. There is a need for novel biomarkers in the management of GE cancers, to yield predictive response to the available therapies. Our study aims to identify leading genes that are differentially regulated in patients with these cancers. We explored the expression data for those genes whose protein products can be detected in the plasma using the Cancer Genome Atlas to identify leading genes that are differentially regulated in patients with GE cancers. Our work predicted several candidates as potential biomarkers for distinct stages of GE cancers, including previously identified CST1, INHBA, STMN1, whose expression correlated with cancer recurrence, or resistance to adjuvant therapies or surgery. To define the predictive accuracy of these genes as possible biomarkers, we constructed a co-expression network and performed complex network analysis to measure the importance of the genes in terms of a ratio of closeness centrality (RCC). Furthermore, to measure the significance of these differentially regulated genes, we constructed an SVM classifier using machine learning approach and verified these genes by using receiver operator characteristic (ROC) curve as an evaluation metric. The area under the curve measure was > 0.9 for both the overexpressed and downregulated genes suggesting the potential use and reliability of these candidates as biomarkers. In summary, we identified leading differentially expressed genes in GE cancers that can be detected in the plasma proteome. These genes have potential to become diagnostic and therapeutic biomarkers for early detection of cancer, recurrence following surgery and for development of targeted treatment.

## Introduction

Cancers of the stomach and esophagus or gastro-esophageal (GE) cancers represent a highly aggressive disease and are one of the major causes of cancer-related death in the world. Stomach cancer is the fifth most common cancer and the third leading cause of cancer-related death worldwide. For example, in 2018, more than 1 million new cases of stomach cancer were diagnosed and about 783,000 people die from it^1^. Likewise, esophageal cancer is the seventh most common cancer and the sixth leading cause of cancer-related death. Each year more than 500,000 new cases of esophageal cancer are diagnosed and about 509,000 people die from it^1^. Despite improvements in surgical and radiation treatments and the availability of newer agents, the prognosis of patients with recurrent GE cancers remains very poor^2–4^. The need for novel strategies to improve current therapy is therefore vital in the management of GE cancers.

It is well known that cancer development and progression are triggered by altered activities and dysregulated expression of genes that control cell proliferation and differentiation^5^. Comparative assessment of genetic aberrations between cancerous and matched normal tissues as control, has facilitated identification of new biomarkers that may also serve as new therapeutics targets or predict various cancer-related outcomes. There are several known biomarkers that are associated with tumorigenesis or have prognostic and predictive values in patients with stomach and esophageal cancers^6–10^. For example, approximately 20% of stomach and gastroesophageal junction cancers are associated with the amplification of the HER2 gene that is an important therapeutic target and predicts response to trastuzumab^8^. Most biomarkers such as TP53 or CDH1, however, have limited therapeutic and predictive values and presence or absence of them does not alter treatment strategies^9^. There is a strong unmet need for novel biomarkers in the management of GE cancers to identify new therapeutic targets and to yield predictive response to the available therapies.

We conducted this study by intersecting the gene expression profiles from The Cancer Genome Atlas (TCGA) with the plasma proteome databases to identify leading genes that are differentially expressed (upregulated or downregulated) in patients with esophageal and stomach cancers^11,12^. We also applied machine-learning approaches to test our predictive accuracy. Overall, the purpose of these analyses is to identify differentially expressed novel tumor-specific genes that code for the plasma proteins and use this information to develop blood-based prognostic biomarker studies in the near future.

## Methods

### TCGA gene expression analyses

We obtained the level-3 HiSeq RSEM gene-normalized RNA-seq gene expression data for stomach adenocarcinoma (STAD) and esophageal carcinoma (ESCA) from the TCGA database^11^. Overall, gene expression data for 415 independent tumor samples and 35 matching normal tissue samples for STAD and for 185 ESCA cases with 11 matching normal tissue samples were available. We also downloaded the plasma proteome database from http://www.plasmaproteomedatabase.org. The database contained information on 1241 protein-coding genes, while gene expression profiles from TCGA mapped to 1232 protein-coding genes. To analyse plasma proteome genes, we used non-parametric Mann–Whitney-U test to identify genes that are expressed at significantly different levels (p < 0.05) in cancerous and normal tissues. The deregulated genes were grouped according to tumor stages based on the available patient data. This allowed identification of genes with expression significantly increased or decreased at multiple stages of cancer.

### Computational method using gene co-expression network analysis

Gene co-expression networks were used for analyzing the importance of genes and their relationships with other genes. In a weighted gene co-expression networks (WGCN), nodes represent the gene expression profiles, edges represent the pairwise correlation between gene expressions while the edge weights represented the correlation strengths. For our study, the correlation strength of a pair of genes was measured by their similarity, which was calculated by the Pearson correlation coefficient (PCC) between their expression profiles. Specifically, for each pair of genes ${g}_{i}$ and ${g}_{j}$, its strength was calculated as

$${S}_{ij}=pcc\left( {g}_{i},{g}_{j}\right)= \frac{covar({x}_{i}, {x}_{j})}{var\left({x}_{i}\right)var({x}_{j})}$$

where ${x}_{i}$ and ${x}_{j}$ are expression profiles of genes ${g}_{i}$ and ${g}_{j}$, respectively; $var\left({x}_{i}\right)$ and $var\left({x}_{j}\right)$ are the variance of ${x}_{i}$ and ${x}_{j}$, respectively while $covar({x}_{i}, {x}_{j})$ is the covariance of ${x}_{i}$ and ${x}_{j}$. Since the result of PCC has a value between − 1 and 1, we transformed the similarity measure ${S}_{ij}$ into $Dissimilarity{\_cor}_{ij}$ and ${Simmilarity\_cor}_{ij}$, as follows:

$${Dissimilarity\_cor}_{ij}=\frac{1-pcc( {g}_{i},{ g}_{j})}{2}$$

$${Similarity\_cor}_{ij}=\frac{1+pcc( {g}_{i},{ g}_{j})}{2}$$

${Similarity\_cor}_{ij}$ represents the positive correlation between the genes since the larger value indicates the stronger positive correlation between the pair of genes, while ${Dissimilarity\_cor}_{ij}$ represents the negative correlation between a pair of genes since the larger value indicates the stronger negative correlation between genes, which is also called the distance of a pair of genes. Both $Dissimilarity{\_cor}_{ij}$ and ${Simmilarity\_cor}_{ij}$ take on values in [0, 1], that was used for further network analysis.

We used WGCNA R package^13^, that is commonly used in recent studies^14,15^, to construct the weighted network for the stomach and esophageal cancer datasets. To filter out the noisy edges in WGCNs, we applied the soft thresholding scheme^13^ by raising the co-expression similarity to a soft power $\beta$ to shrink the lower correlations. Hence, the strengths in WGCN is represented by

$${Dissimilarity\_cor}_{ij}={\left(\frac{1-pcc\left( {g}_{i},{ g}_{j}\right)}{2}\right)}^{\beta }$$

$${Similarity\_cor}_{ij}={\left(\frac{1+pcc\left( {g}_{i},{ g}_{j}\right)}{2}\right)}^{\beta }$$

where $\beta \ge 1.$ The criterion for the determination of the soft power $\beta$ is dependent on the model fitting index of the scale-free topology^13^. The scale-free networks were constructed because they have strong ability to tolerate against errors^16^. We then removed the self-loop edges of nodes by setting the diagonal elements of the adjacency matrix as 0. Also, we applied a hard threshold to remove weak edges between nodes by setting the threshold as 0.01.

Next, we calculated the closeness centrality measures to identify the closeness of a particular node to all other nodes in a network^17^. This closeness centrality is the inverse of the average shortest-path distance from one node to other nodes in the network. It also indicates the efficiency of one node to spread information through a network. Finally, based on the closeness centrality score of genes in networks, we defined a novel metric of gene importance in networks as the ratio of the closeness centrality (RCC) of a gene in its corresponding similarity network and its dissimilarity network, i.e.

$$RC{C}_{N}=\frac{{CN}_{sim}}{{CN}_{dis}} \quad \mathrm{and}\quad RC{C}_{P}=\frac{{CT}_{sim}}{{CT}_{dis}}$$

where ${CN}_{sim}$ and ${CN}_{dis}$ represent the closeness centrality score in the similarity network and the dissimilarity network of normal samples, while ${CT}_{sim}$ and ${CT}_{dis}$ represent the closeness centrality score in the similarity network and the dissimilarity network of tumor samples, respectively. We expect that the biomarkers should be significantly different in terms of log2(RCC) between the normal and tumor samples.

### Machine learning approach to test the significance

To test the significance of the differentially regulated genes, we constructed a support vector machine (SVM) classifier with linear kernel. The features were based on the leading upregulated genes, the leading downregulated genes and a set of randomly selected genes for each cancer type. Accordingly, we used Receiver Operator Characteristic (ROC) curve as the evaluation metric for the classification of cancer patients and normal samples as in previous studies^18^. The ROC curve is an evaluation metric for binary classification problems, which visualizes the trade-off between true positive rate (TPR) and false positive rate (FPR). We then measured the area under the curve (AUC), as higher the AUC, the better the performance of the model in distinguishing between normal and tumor samples.

## Results

### Leading upregulated genes in GE cancers

**Figure 1:**
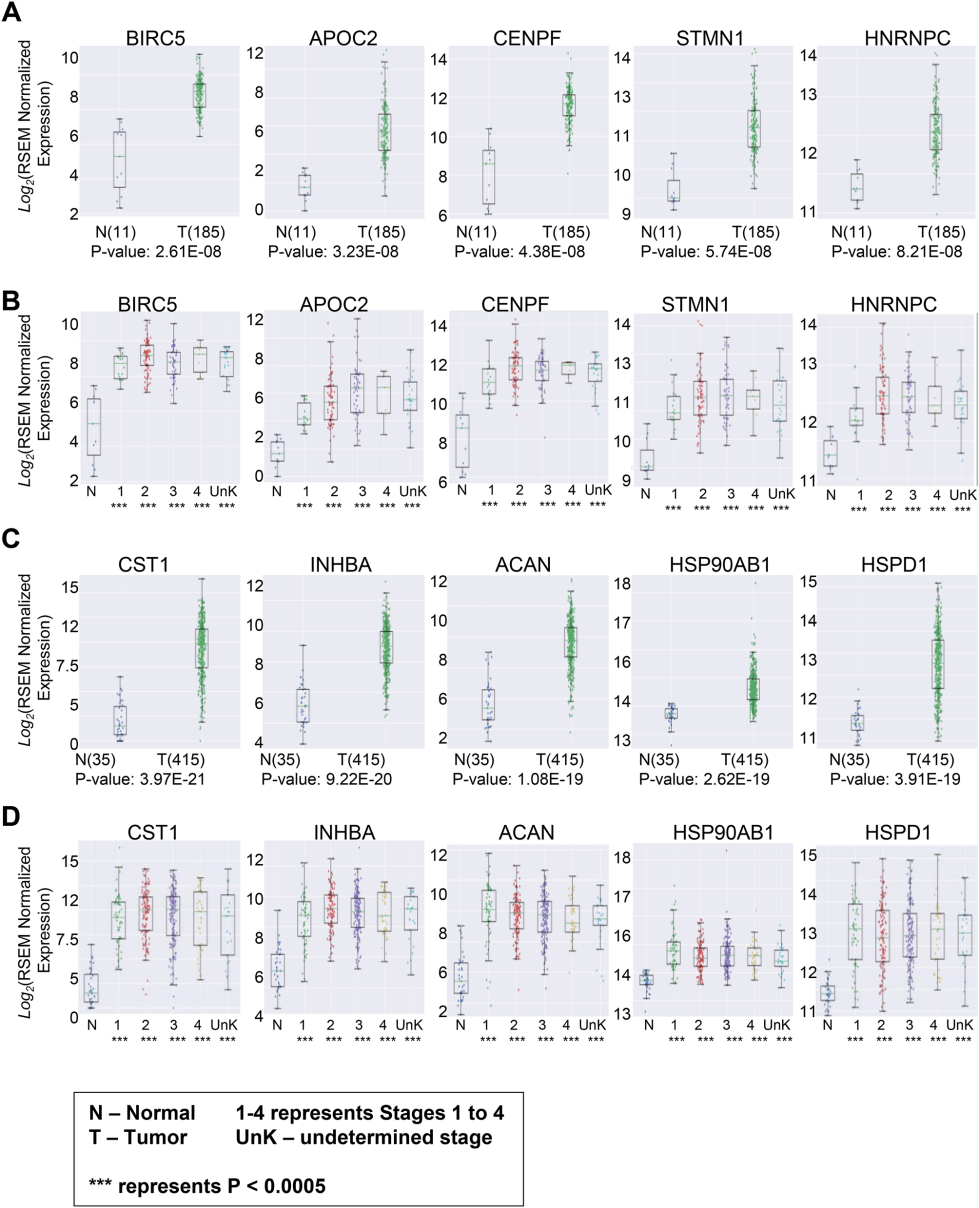
(**A**) The comparison between patients with esophageal cancer versus control individuals showed significant overexpression BIRC5, APOC2, CENPF, STMN1, and HNRPC in patients with esophageal cancer. (**B**) BIRC5, APOC2, CENPF, STMN1, and HNRPC significantly overexpressed across all stages in patients with esophageal cancer. (**C**) The comparison between patients with stomach cancer and healthy control showed significant overexpression of CST1, INHBA, ACAN, HSP0AB1, and HSPD1 in patients with stomach cancer. (**D**) Assessment of CST1, INHBA, ACAN, HSP90AB1, and HSPD1 in different stages of stomach cancers showed a significant overexpression of these genes across all stages in patients with stomach cancer.

We examined gene expression of 1232 protein-coding genes that were detected in plasma proteome in tumors of 185 patients with esophageal cancer and in the matching tissue of 11 subjects with no cancer. Among cancer patients, 18 (9.7%) had stage I tumors, 78 (42.2%) had stage II disease, 56 (30.3%) had stage III disease, and 9 (4.9%) had stage IV cancer. In 24 (13%) patients, cancer stage was not known. The comparison between esophageal tumors and healthy tissue showed BIRC5 (p = 2.61E−08), APOC2 (p = 3.23E−08), CENPF (p = 4.38E−08), STMN1 (p = 5.74E−08), and HNRPC (p = 8.21E−08) to be five leading genes overexpressed in esophageal cancer (Fig. 1A). The stage-based assessment of overexpressed genes showed significant overexpression of BIRC5, APOC2, CENPF, STMN1, and HNRPC across all cancer stages including early, locally advanced and metastatic esophageal tumors (Fig. 1B). The significance of expression for each gene compared between normal and tumor samples (p values), along with the number of samples in each stage are provided in Supplementary Table S1.

For stomach cancer, we evaluated 415 cases and compared them with 35 normal tissue samples. Among patients with stomach cancer, 57 (13.7%) had stage I cancer, 123 (29.6%) had stage II disease, 169 (40.7%) had stage III disease, and 41 (9.9%) had stage IV cancer. In 25 patients (6%) with stomach cancer, the disease stage was not known. Comparison between normal stomach tissue and stomach tumors showed that CST1 (p = 3.97E−21), INHBA (p = 9.22E−20), ACAN (p = 1.08E−19), HSP90AB1 (p = 2.62E−19), and HSPD1 (p = 3.91E−19) were the leading five genes that were overexpressed in stomach cancer (Fig. 1C). The stage-based assessment of overexpressed genes showed significant upregulation of CST1, INHBA, ACAN, HSP90AB1, and HSPD1 genes across all stages, including early, locally advanced and metastatic stomach cancer (Fig. 1D). The significance of expression for each gene compared between normal and tumor samples (p values), along with the number of samples in each stage are provided in Supplementary Table S2.

### Stage-specific upregulation of genes in GE cancers

**Figure 2:**
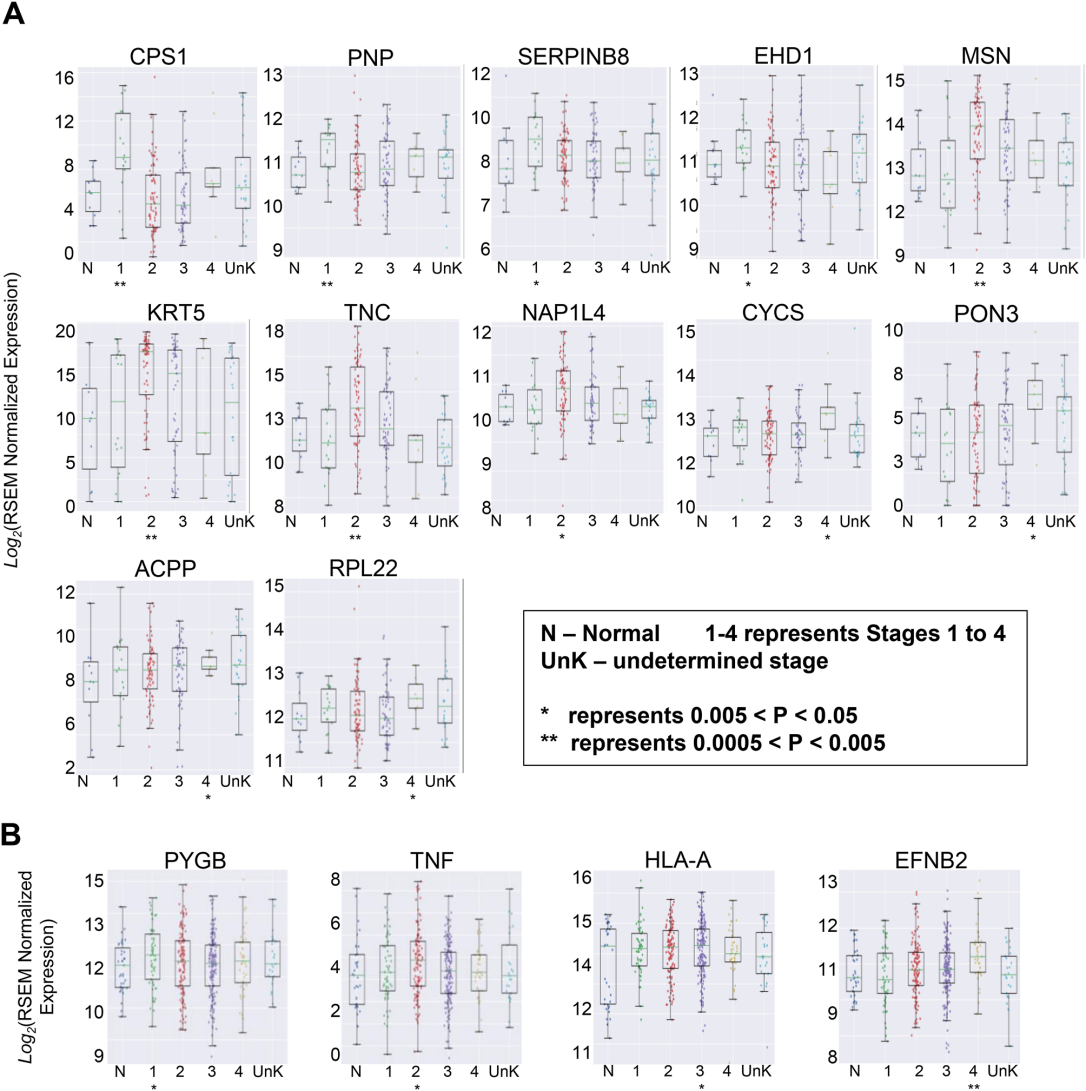
(**A**) Stage-based analysis of esophageal cancer showed a differential expression of various genes based on the stage of the disease. (**B**) Stage-based analysis showed a differential expression of various genes based on the stage of the disease in patients with stomach cancer.

We next examined the pattern of gene expression based on the specific-stage of the disease in GE cancers. In addition to the five overexpressed genes reported above, the stage-based analysis showed a differential expression of following genes based on the stage of the disease. Patients with stage I esophageal cancer had significantly higher expression of CPS1 (p = 0.003), PNP (p = 0.007), SERPINB8 (p = 0.042) and EHD1 (p = 0.046). In patients with stage II disease, MSN (p = 0.003), KRT5 (p = 0.004), TNC (p = 0.007), and NAP1L4 (p = 0.018) were overexpressed compared with other stages of the disease. In patients with stage IV esophageal cancer CYCS (p = 0.014), PON3 (p = 0.14), ACPP (p = 0.047), and RPL22 (p = 0.047) were significantly upregulated compared with patients with early-stage cancer. We did not notice a stage-specific upregulated gene in stage III esophageal cancer (Fig. 2A). The significance of expression for each gene compared between normal and tumor samples (p values), are provided in Supplementary Table S3. Likewise, the stage-based analysis in patients with stomach cancer showed a differential expression of several genes at the specific stages of the disease. Thus, patients with stage I, II, III, and IV stomach cancer have significantly higher overexpression of PYGB (p = 0.043), TNF (p = 0.02), HLA-A (0.05), and EFNB2 (0.001) genes, respectively (Fig. 2B). The significance of expression for each gene compared between normal and tumor samples (p values), are provided in Supplementary Table S4.

### Leading progressively upregulated genes in GE cancers

**Figure 3:**
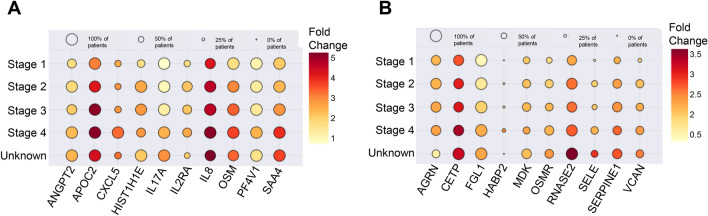
(**A**) Stage-wise incremental over-expressions of leading genes in patients with esophageal cancer. (**B**) Stage-wise incremental over-expressions of leading genes in patients with stomach cancer.

We also examined if certain gene expression pattern intensifies in parallel with the progression of the disease. This analysis showed a gradual stage-wise increasing expression pattern for ANGPT2, APOC2, CXCL5, HIST1H1E, IL17A, IL2RA, IL8, OSM, PF4V1, and SAA4 in esophageal cancer. Among them, a gradual increase from early-stage cancer to more advanced stages were strongest for APOC2 and IL8 genes followed by for SAA4 and OSM genes, whereas HIST1H1E, PF4V1, CXCL5, and IL2RA expression showed limited stage-wise upregulation (Fig. 3A). In the stomach cancer, the stage-wise analysis showed a gradual increasing expression pattern for AGRN, CETP, FGL1, HABP2, MDK, OSMR, RNASE2, SELE, SERPINE1, and VCAN. Among them, the upregulation from the early-stage cancer to more advanced stages were the strongest for RNASE2, SERPINE1, and CETP (Fig. 3B).

### Leading downregulated genes in GE cancers

**Figure 4:**
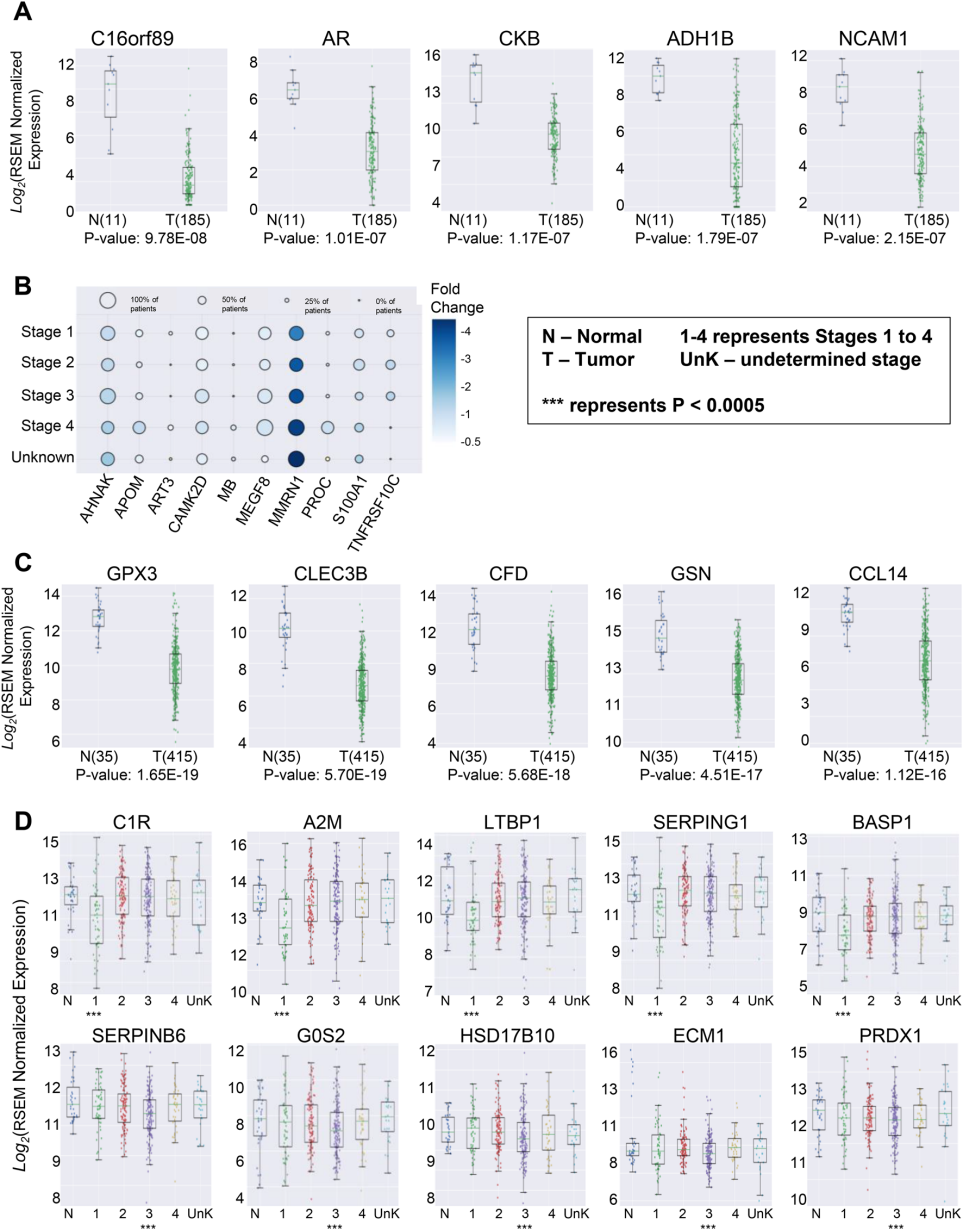

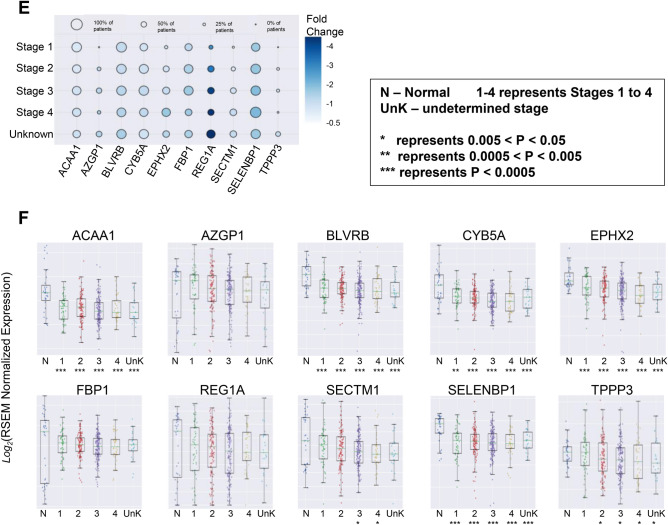
(**A**) Comparison between healthy control and patients with esophageal cancer showed that C16orf89, AR, CKB, ADH1B, and NCAM1 were the top five genes that were significantly down regulated in patients with esophageal cancer. (**B**) Stage-wise incremental downregulation of leading genes in patients with esophageal cancer. (**C**) Comparison between healthy control and patients with stomach cancer showed that: GPX3, CLEC3B, CFD, GSN, and CCL14 were the top five genes that were significantly down regulated in patients with stomach cancer. (**D**) The stage-based analysis showed a differential downregulation of various genes based on the stage of the disease. (**E**) Stage-wise incremental downregulation of leading genes in patients with stomach cancer. (**F**) Stage-wise downregulation of leading genes in patients with stomach cancer.

In addition to the upregulated genes, we also examined leading downregulated genes that could play a major role in pathogenesis and progression of cancer. Our analysis showed that following five genes were most significantly downregulated in esophageal cancer: C16orf89 (9.78E−08), AR (1.01E−07), CKB (1.17E−07), ADH1B (1.79E−07), and NCAM1 (2.15E−07) (Fig. 4A). We did not observe any stage-specific down-regulation for esophageal cancer. However, the stage-wise analysis showed a gradual downregulation of the following genes from stage I to IV esophageal cancer: AHNAK, APOM, ART3, CAMK2D, MB, MEGF8, MMRN1, PROC, S100A1, and TNFRSF10C (Fig. 4B).

In patients with stomach cancer following five genes were most significantly downregulated: GPX3 (1.65E−19), CLEC3B (5.70E−19), CFD (5.68E−18), GSN (4.5 IE−17), and CCL14 (1.12E−16) (Fig. 4C). The stage-based analysis showed that C1R (2.34E−04), A2M (3.15E−04), LTBP1 (3.15E−04), SERPING1 (4.23E−04), and BASP1 (4.88E−04) were significantly downregulated only in patients with stage I (early-stage) stomach cancer whereas SERPINB6 (2.0E−03), G0S2 (1.80E−02), HSD17B10 (2.50E−02), ECM1 (2.60E−02), and PRDX1 (2.80E−02) were significantly downregulated in patients with stage III (locally advanced) stomach cancer (Fig. 4D). The significance of expression for each gene compared between normal and tumor samples (p values), are provided in Supplementary Table S5. The stage-wise analysis also revealed a progressive downregulation of the following genes from stage I to IV stomach cancer: ACAA1, AZGP1, BLVRB, CYB5A, EPHX2, FBP1, REG1A, SECTM1, SELENBP1, and TPPP3 (Fig. 4E,F). The significance of expression for each gene compared between normal and tumor samples (p values), are provided in Supplementary Table S6.

### Measuring robustness and significance of the identified biomarkers

**Figure 5:**
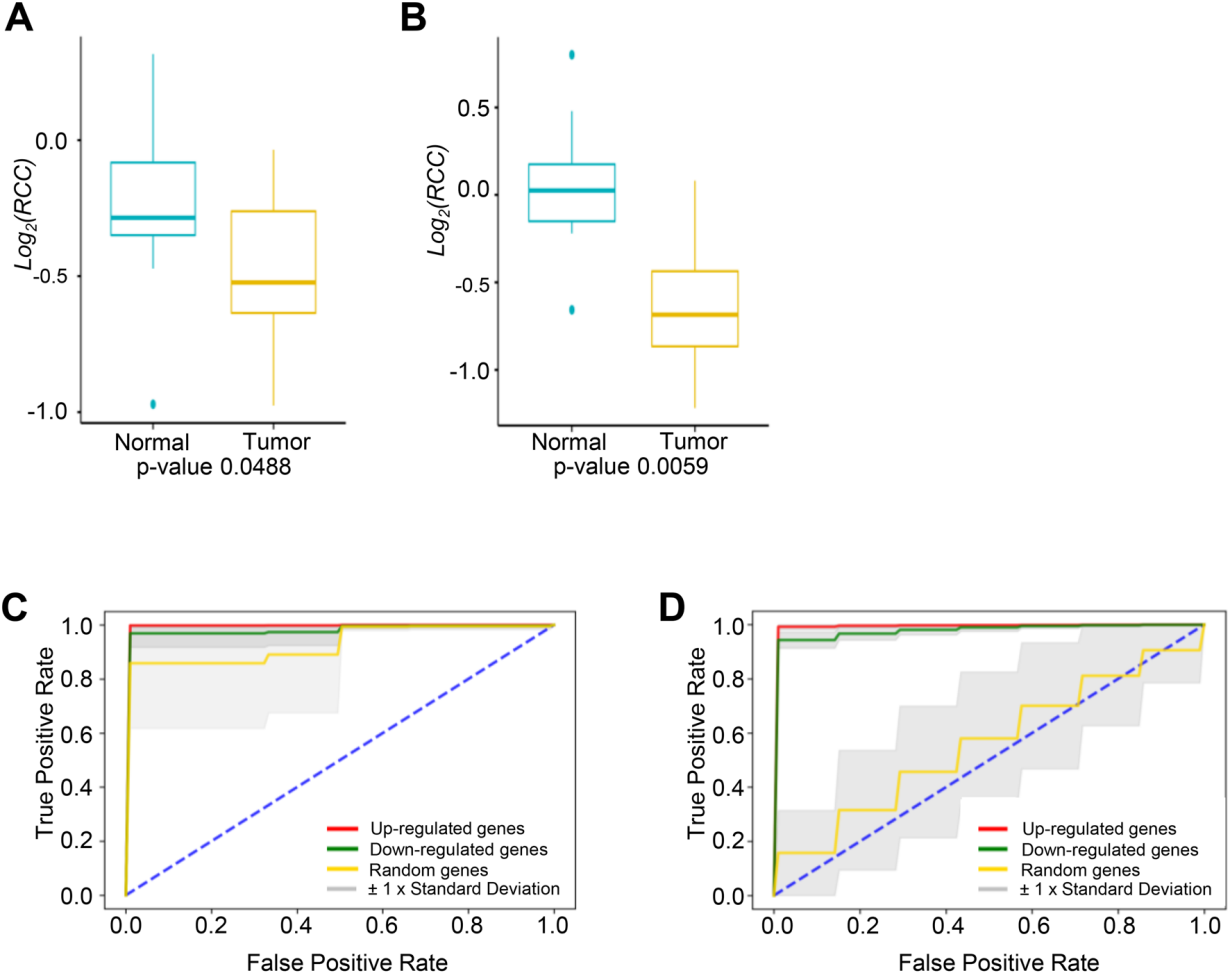
(**A**) The log ratio of closeness centrality (RCC) between normal and tumor samples for the proposed potential biomarkers in Esophageal cancer. (**B**) The log ratio of closeness centrality (RCC) between normal and tumor samples for the proposed potential biomarkers in Stomach cancer. (**C**) Comparison of ROC curves with features of the leading up-regulated genes, the leading down-regulated genes and the random selected genes for classification of tumor and normal samples in esophageal cancer. The dashed blue line in the diagonal presents the ROC curve of a random predictor, which has an AUC of 0.5 and can be used as the baseline to validate the effectiveness of our models. The mean AUC scores of the SVM models based on the up-regulated genes and the down-regulated genes are 0.9941 (standard deviation: 0.0031) and 0.9788 (standard deviation: 0.0265), respectively. The mean AUC of the comparison group, which uses the random selected genes, has the lowest score 0.9280 (standard deviation: 0.1137). (**D**) Comparison of ROC curves with features of the leading up-regulated genes, the leading down-regulated genes and the random selected genes for classification of tumor and normal samples in stomach. The dashed blue line in the diagonal presents the ROC curve of a random predictor, which has an AUC of 0.5 and can be used as the baseline to validate the effectiveness of our models. The mean AUC scores of the SVM models based on the up-regulated genes and the down-regulated genes are 0.9924 (standard deviation: 0.0038) and 0.9770 (standard deviation: 0.0114), respectively. The mean AUC of the comparison group, which uses the random selected genes, has the lowest score 0.5603 (standard deviation: 0.1664).

We next sought to evaluate the predictive accuracy of the leading candidates as potential biomarkers in GE cancers. Towards this, we calculated the similarity and dissimilarity matrices. These matrices were used to construct the co-expression network with weighted co-expression network analysis (WGCNA)^13^. Using complex network analysis, we calculated the centrality of the genes in the constructed network and verified the importance of biomarkers. The log RCC calculated for the differentially expressed genes between normal and tumor samples are shown in Fig. 5A,B for esophageal and stomach cancers, respectively. To statistically confirm our conclusion, we applied the paired samples Wilcoxon signed-rank test to log_2_(RCC) of the identified biomarkers between normal and tumor samples for both cancer types and found them to be significant (p < 0.05). Based on these results, we are confident on our predictive accuracy of these genes as potential biomarkers.

Since the sample sizes of the esophageal and stomach cancer datasets were small, a fivefold cross validation was adopted to evaluate the performances of SVM models on each dataset. The corresponding mean ROC curves of 50 executions of fivefold cross validation are illustrated in Fig. 5C,D. For the esophageal cancer dataset, the feature sets include the up-regulated genes (Fig. 1A), the down-regulated genes (Fig. 4A) and five randomly selected genes. Similarly, for the stomach cancer dataset, the feature sets include the up-regulated genes (Fig. 1C), the down-regulated genes (Fig. 4C) and five randomly selected genes as the third set. The model based on the up-regulated gene group has the AUC of 0.9941 with standard deviation of 0.0031 for esophageal cancer and the AUC of 0.9924 with standard deviation of 0.0038 for stomach cancer. Likewise, the AUC of the downregulated genes are 0.9788 (standard deviation 0.0265) and 0.9770 (standard deviation 0.0114) for esophageal and stomach cancers respectively. Meanwhile, the classifier model using the random selected genes has the lowest AUC score 0.9280 with standard deviation of 0.1137 for esophageal cancer and 0.5603 with standard deviation of 0.1664 for stomach cancer. This suggests that the features based on the differentially expressed genes are significant at identifying patients from normal samples compared with randomly selected genes. Specifically, the AUC scores of both the esophageal and stomach cancer using the up-regulated genes are greater than 0.99, which illustrates our proposed biomarkers have strong capability at differentiating the class of cancer patient samples from normal samples.

## Discussion

Our investigation identified leading genes that are upregulated or downregulated in patients with GE cancers. We specifically focussed on those genes whose protein products can be detected in the plasma, as measured in the plasma proteome database. Thus, our investigation has a direct translational impact. The abnormal gene expression plays a pivotal role in tumor development and progression^5^. We noted that compared to normal tissue, BIRC5, CENPF, STMN1, APOC2, and HNRPC were the five most significantly upregulated genes in esophageal cancer. Furthermore, these genes were also overexpressed in stomach cancer.

The baculoviral IAP repeat containing 5 (BIRC5) gene, also known as survivin, is a member of the inhibitor of apoptosis (IAP) family, where it encodes regulatory proteins that prevent apoptotic cell death. Survivin localizes to the mitotic spindle and participates in regulating mitosis. In addition to GE cancers, it is highly expressed in various other malignancies and is associated with poor outcomes including a shorter survival period^19–21^. CENPF gene encodes centromere protein F that associates with the centromere–kinetochore complex. CENP-F protein is thought to be a cell cycle regulated protein that may play a role in chromosome segregation during mitosis. Interestingly, there is evidence that CENPF expression is associated with inferior outcomes in patients with esophageal cancer and patients with lower CENPF expression had a better survival rate compared with those with higher CENPF expression^22^. The CENPF gene is also amplified in other solid tumors including hepatocellular and breast cancers and correlates with patients’ outcomes^23–25^. As cancer cells undergo active division, perhaps the up regulation of genes like BIRC5, CENPF could be a direct consequence of active mitosis.

STMN1 belongs to the stathmin family of genes and encodes a cytosoplasmic phosphoprotein stathmin 1. The encoded protein belongs to the family of microtubule-destabilizing proteins that control the assembly and disassembly of the mitotic spindle and thereby, regulate mitosis. Similar to esophageal cancer, STMN1 is highly expressed in various cancers, including leukemia, breast, prostate and lung cancer, and is a promising target for cancer therapy^26,27^. There is some evidence that it may have a prognostic significance in the early-stage gastric cancer. For example, a study that evaluated STMN1 role in both operable and advanced gastric cancers showed that in the operable cohort, STMN1 expression correlated with cancer recurrence, and resistance to adjuvant therapies^28^.

In contrast to the relatively known roles of BIRC5, CENPF, and STMMN1 in malignancies, functions of APOC2 and HNRNPC genes in cancer cell are less well defined. The APOC2 gene encodes a lipid-binding protein that belongs to the apolipoprotein family and is a component of the very low-density lipoprotein. This protein activates the enzyme lipoprotein lipase, which hydrolyzes triglycerides. APOC2 mutations could cause hyperlipoproteinemia type IB, characterized by hypertriglyceridemia, xanthomas, and early atherosclerosis^29,30^. HNRNPC gene encodes a protein that belongs to the subfamily of ubiquitously expressed heterogeneous nuclear ribonucleoproteins (hnRNPs). The hnRNPs are RNA binding proteins and are associated with pre-mRNAs in the nucleus. These proteins are involved in pre-mRNA processing and other aspects of mRNA metabolism and transport along with cell proliferation and differentiation^31^. However, functions of hnRNPs in tumorigenesis and cancer progression in solid and hematological malignancies are not well understood^32^.

Our analysis showed that compared with normal stomach tissue, CST1, INHBA, ACAN, HSP90AB1, and HSPD1 were the leading five genes that were overexpressed in stomach cancer. These genes were also upregulated in patients with esophageal cancer. The CST1 gene encodes a secretory peptide called Cystatin SN, which is a cysteine proteinase inhibitor. Cysteine proteases are involved in tissue remodeling during development, and they support the migration of cancer cells. CST1 itself is known to promote proliferation, clone formation, and metastasis in breast cancer cells and high CST1 expression is negatively correlated with breast cancer survival^33^. CST1 has also been considered as a potential tumor marker in various epithelial malignancies^33,34^. Of note, a study involving patients with esophageal squamous cell carcinoma whose tumors express high levels of Cystatin SN showed favorable survival compared with those patients with low Cystatin SN expression^35^. Inhibin-βA (INHBA), a ligand belonging to the transforming growth factor-β superfamily, is associated with cell proliferation in cancer. INHBA is overexpressed in various types of cancers including esophageal and stomach tumors^36,37^. Overexpression of the INHBA gene is considered a useful independent predictor of outcomes in patients with gastric cancer after the curative surgery. High INHBA gene expression has shown to be associated with significantly poorer 5-year overall survival compared with low expression cases in patients with stomach cancer^37^. HSPD1 and HSP90AB1 belong to heath shock protein (HSP) group and encodechaperonin family proteins^38^. HSPD1 encodes a mitochondrial protein, which is important for assembly of imported proteins in the mitochondria and may function as a signaling molecule in the immune system. HSP90AB1 is thought to play a role in gastric apoptosis and inflammation. HSPs control a wide variety of signaling and cellular responses and have been classified into several subfamilies such as the HSP60s, HSP70s, HSP90s, and HSP100s^39^. HSP expression often correlates with patient prognosis in various malignancies^40–42^ For example, HSP60 has been identified as an independent prognostic factor for both overall survival and recurrence-free survival in patients with early-stage stomach cancer^42^. The ACAN gene is a member of the aggrecan/versican proteoglycan family. The encoded protein is an integral part of the extracellular matrix in cartilaginous tissue, and it withstands compression in cartilage. Mutations in this gene may be involved in skeletal dysplasia and spinal degeneration, however, its role in cancer is not well understood.

With respect to downregulated genes, C16orf89, AR, CKB, ADH1B, and NCAM1 were the leading downregulated genes in patients with esophageal cancer. C16orf89 is predominantly expressed in the thyroid gland and is involved in the development and function of the thyroid^43^. Its role in tumorigenesis and progression has not been elucidated yet. The androgen-receptor (AR) gene encodes AR. Once AR binds its hormone ligand testosterone, it translocates into the nucleus, and stimulates transcription of androgen responsive genes^44^. In vitro evidence suggests a significant influence of sex hormones upon cancer growth^44,45^. For example, AR pathway plays an important role in the development of prostate cancer and various other epithelial malignancies including bladder, kidney, lung, breast, liver and ovary^45^. However, AR role in GE cancers development and progression is not known^46^. CKB or creatinine kinase B gene encodes a cytoplasmic enzyme that is involved in energy homeostasis. Its dysregulation could promote cancer invasiveness and progression^47^. Similar to AR, its disease modulating effect in GE cancers is unknown. ADHIB encodes alcohol dehydrogenase 1B enzyme. Evidence suggests that genetic polymorphisms of this enzyme has been associated with the increased risk of the aerodigestive cancer triggered by alcohol consumption^48^. The NCAM1 gene encodes a cell adhesion protein, a member of the immunoglobulin superfamily that is involved in both cell to cell and cell to matrix interactions. Its downregulation has been linked to cancer progression and development of metastases in gastrointestinal and other malignancies^49^.

In patients with stomach cancers, GPX3, CLEC3B, CFD, GSN, and CCL14 were the leading five genes that were most significantly downregulated. GPX3 encodes glutathione peroxidase that belongs to a family of selenocysteine-containing redox enzymes that play important roles in cell signaling and immune modulation^50^. Consistent with our observation of its downregulation, promoter hypermethylation and downregulation of GPX3 in melanoma, stomach, head and neck, cervical and lung cancers suggest that GPX3 serves as a tumor suppressor in these cancers^50,51^. C-Type Lectin Domain Family 3 Member B (CLEC3B) is a member of the C-type lectin superfamily that encodes tetranectin. Dysregulation of CLEC3B has been reported in various epithelial cancers including stomach cancer^52,53^. Chen and others using TCGA database also noted downregulation of CLEC3B in stomach cancer. However, when they evaluated 328 patients with early-stage stomach cancer, high intratumoral tetranectin level was significantly associated with tumor invasion, lymph node metastasis, advanced TNM stage, and a shorter overall survival^53^. CFD or complement factor D encodes a serine protease that catalyze breakdown of factor B a rate limiting step of alternative pathway of complement activation. Impaired balance of complement activation could promote inflammation and tumorigenesis resulting in malignant cells proliferation, migration, invasiveness and metastasis^54,55^**.** GSN or Gelsolin gene encodes a protein that is involved in assembly and disassembly of actin filaments. Gelsolin has been attributed in prostate tumorigenesis and malignant transformation^56,57^. The C–C type chemokine 14 gene is known to induce targeted cell migration and is thought to play a role in carcinogenesis and metastasis of certain malignancies including breast cancer^58,59^.

Aside from the leading upregulated and downregulated genes in patients with GE cancers, we also noted a stage-wise upregulation of several genes, such as APOC2, IL8, RNASE2, SERPINE1, and CETP, and stage-wise downregulation of other genes that play important role in and survival, including AHNAK, MEGF8, MMRN1, PROC, REG1A, SECTM1, TNFRSF10C, and TPPP3. The stage-related expression of these genes suggests their potential role in the disease progression and utility as monitoring markers or therapeutic targets. The cholesteryl ester transfer protein (CETP) for example maintains cholesterol homeostasis and has been identified as a potential target for estrogen positive breast cancer^60^. Conversely, AHNAK can act as a tumour suppressor gene and mediates the negative regulation of cell growth^61^.

Furthermore, we also evaluated the predictive accuracy and the significance of the genes we identified. In recent years, deep neural networks have achieved enormous successes for such applications^62–64^. However, deep learning networks was not adopted in this study. This is primarily because, deep learning algorithms are non-linear and normally has millions of parameters^65^. Since the aim of our study is to identify key biomarkers for GE cancers, the explanation of model is crucial to evaluate the significance of genes. Contrarily, the classical machine learning models, such as linear models, provide a direct relationship between features and their prediction which makes it relatively straightforward to reason the decision mechanism of the model. Also, to avoid overfitting, more data are needed for the training of deep learning models. Moreover, the variations of the training data are necessary to construct a robust model. As the dataset sizes are not large in this study, the deep learning model will result in overfitting, if we apply deep learning to these datasets.

While our work provides a significant amount of novel information regarding the behavior of cancer-related molecules in GE cancers, it does not assess the level of gene expression based on the molecular classification of stomach and esophageal cancers^7^. Furthermore, we did not have information on histopathology of these cancer types and therefore, were not able to segregate the data based on histopathology. Finally, while we examine the up/down regulated genes, solely from the perspective of their differential expression, it will be interesting to investigate these candidates in cohorts of immunodeficient patients as it will provide additional knowledge on how these candidates may promote adaptive alterations of host gut- and tissue-based microbiome^66^. In summary, the present study identified leading upregulated and downregulated genes in GE cancers. Since expression of the upregulated genes was minimal in both stomach and esophageal normal tissues, these genes have a strong potential to become diagnostic and therapeutic biomarkers for screening and early detection of cancer, recurrence following surgery and for anti-cancer therapies. Future studies will be required for validating diagnostic, therapeutic and prognostic importance of these genes. Our group plans to prospectively evaluate prognostic and predictive values of selected genes in a cohort of patients with metastatic gastroesophageal cancer who are treated with combination chemotherapy.

## Supplementary Information

Supplementary Information 1.
