# Supplementary material for: Mining the plasma-proteome associated genes in patients with gastro-esophageal cancers for biomarker discovery
Source: Sci Rep. 2021 Apr 7;11:7590. doi: 10.1038/s41598-021-87037-w (PMC8027878; doi:10.1038/s41598-021-87037-w)
Supplement: Supplementary file 1 — Supplementary Information 1. [file 41598_2021_87037_MOESM1_ESM.pdf]

# Mining the Plasma-Proteome Associated Genes in Patients with Gastro-Esophageal Cancers for Biomarker Discovery

Frederick S. Vizeacoumar[1], Hongyu Guo[2], Lynn Dwernychuk[3], Adnan Zaidi[3,4], Andrew Freywald[1], Fang-Xiang Wu[2,5,6], Franco J. Vizeacoumar[3,4$] and Shahid Ahmed[3,4,$]

[1]Department of Pathology and Laboratory Medicine, College of Medicine, University of Saskatchewan, Saskatoon, Canada, [2]Division of Biomedical Engineering, University of Saskatchewan, Saskatoon, Canada. [3]Cancer Research, Saskatchewan Cancer Agency, Saskatoon, Canada, [4]Division of Oncology, University of Saskatchewan, Saskatoon, Canada, [5]Department of Mechanical Engineering, University of Saskatchewan, Canada, [6]Department of Computer Science, University of Saskatchewan, Canada.

**Short Title:** Predictive biomarkers for gastro-esophageal cancers

**Key words:** Stomach cancer, esophageal cancer, gastroesophageal cancer, biomarkers, plasma proteome

**$ Correspondence to:**

Shahid Ahmed, MD, PhD, FRCPC, FACP
Saskatoon Cancer Center
University of Saskatchewan
20 Campus Drive
Saskatoon, SK
Canada, S7N4H4
Ph: 306-655-2710
Fax: 306-655-0633
Email: shahid.ahmed@saskcancer.ca

Franco J. Vizeacoumar, BSc, PhD
Saskatoon Cancer Center
University of Saskatchewan
107 Wiggins Road
Saskatoon, SK
Canada, S7N5E5
Ph: 306-966-7010
Fax: 306-966-7047
Email: franco.vizeacoumar@usask.ca

# Supplementary Tables

**Supplementary Table 1: P-values for the genes in Figure 1B compared to Normal samples and** number of samples in Normal and each stages of Esophageal Carcinoma (ESCA)

| Esophageal Carcinoma (ESCA) | | | | | | |
|---|---|---|---|---|---|---|
| Gene Symbol | Normal | Stage 1 | Stage 2 | Stage 3 | Stage 4 | Stage Unknown |
| BIRC5 | | 7.21E-06 | 7.95E-08 | 2.38E-07 | 9.85E-05 | 2.10E-06 |
| APOC2 | | 4.77E-06 | 1.55E-07 | 1.67E-07 | 9.85E-05 | 3.48E-06 |
| CENPF | | 2.37E-05 | 9.09E-08 | 3.10E-07 | 9.85E-05 | 5.71E-06 |
| STMN1 | | 1.32E-05 | 1.89E-07 | 2.38E-07 | 1.78E-04 | 7.90E-06 |
| HNRNPC | | 1.04E-04 | 1.55E-07 | 3.39E-07 | 9.85E-05 | 1.09E-05 |
| | | | | | | |
| No. of Samples | 11 | 18 | 78 | 56 | 9 | 24 |

**Supplementary Table 2: P-values for the genes in Figure 1D compared to Normal samples and** number of samples in Normal and each stages of Stomach Adenocarcinoma (STAD)

| Stomach Adenocarcinoma (STAD) | | | | | | |
|---|---|---|---|---|---|---|
| Gene Symbol | Normal | Stage 1 | Stage 2 | Stage 3 | Stage 4 | Stage Unknown |
| CST1 | | 3.69E-15 | 9.10E-19 | 7.93E-19 | 9.89E-13 | 3.51E-09 |
| INHBA | | 7.12E-12 | 5.73E-18 | 3.55E-18 | 1.45E-12 | 1.22E-08 |
| ACAN | | 1.10E-12 | 1.18E-17 | 7.63E-18 | 2.62E-12 | 5.09E-09 |
| HSP90AB1 | | 4.77E-14 | 4.23E-16 | 8.98E-18 | 2.82E-12 | 2.03E-08 |
| HSPD1 | | 2.32E-12 | 8.07E-16 | 4.93E-18 | 1.16E-12 | 4.26E-09 |
| | | | | | | |
| No. of Samples | 35 | 57 | 123 | 169 | 41 | 25 |

**Supplementary Table 3: P-values and** the stages for the genes in Figure 2A compared to Normal samples in ESCA

| Top stage specific up-regulation | | |
|---|---|---|
| Stage | Gene Symbol | P-Value |
| 1 | CPS1 | 0.003 |
| 1 | PNP | 0.007 |
| 1 | SERPINB8 | 0.042 |
| 1 | EHD1 | 0.046 |
| 2 | MSN | 0.003 |
| 2 | KRT5 | 0.004 |
| 2 | TNC | 0.007 |
| 2 | NAP1L4 | 0.018 |
| 4 | CYCS | 0.014 |
| 4 | PON3 | 0.014 |
| 4 | ACPP | 0.047 |
| 4 | RPL22 | 0.047 |

**Supplementary Table 4: P-values and** the stages for the genes in Figure 2B compared to Normal samples in STAD

| Top stage specific up regulation | | |
|---|---|---|
| Stage | Gene | P-Value |
| 1 | PYGB | 0.043 |
| 2 | TNF | 0.02 |
| 3 | HLA-A | 0.05 |
| 4 | EFNB2 | 0.001 |

**Supplementary Table 5: P-values and** the stages for the genes in Figure 4D compared to Normal samples in STAD

| Top stage specific down regulation | | |
|---|---|---|
| Stage | Gene | P-Value |
| 1 | C1R | 2.34E-04 |
| 1 | A2M | 3.15E-04 |
| 1 | LTBP1 | 3.15E-04 |
| 1 | SERPING1 | 4.23E-04 |
| 1 | BASP1 | 4.88E-04 |
| 3 | SERPINB6 | 2.00E-03 |
| 3 | G0S2 | 1.80E-02 |
| 3 | HSD17B10 | 2.50E-02 |
| 3 | ECM1 | 2.60E-02 |
| 3 | PRDX1 | 2.80E-02 |

**Supplementary Table 6: P-values for the genes in Figure 4F compared to Normal** samples in each stages for Stomach Adenocarcinoma (STAD)

| Top progressively down-regulated genes | | | | | |
|---|---|---|---|---|---|
| Gene Symbol | Stage 1 | Stage 2 | Stage 3 | Stage 4 | Stage Unknown |
| ACAA1 | 7.57E-06 | 7.34E-06 | 1.98E-07 | 1.98E-04 | 4.86E-04 |
| AZGP1 | 5.00E-01 | 3.83E-01 | 1.40E-01 | 2.90E-01 | 2.01E-01 |
| BLVRB | 2.66E-06 | 1.02E-07 | 3.66E-09 | 1.60E-05 | 1.03E-05 |
| CYB5A | 2.00E-03 | 2.76E-05 | 1.12E-07 | 2.75E-05 | 8.20E-04 |
| EPHX2 | 6.30E-04 | 2.89E-06 | 1.98E-08 | 5.04E-08 | 1.87E-05 |
| FBP1 | 1.42E-01 | 9.10E-02 | 5.50E-02 | 1.22E-01 | 1.84E-01 |
| REG1A | 2.68E-01 | 1.05E-01 | 5.20E-02 | 1.82E-01 | 1.50E-01 |
| SECTM1 | 1.34E-01 | 5.50E-02 | 1.00E-02 | 3.90E-02 | 6.30E-02 |
| SELENBP1 | 4.19E-06 | 1.14E-07 | 1.20E-08 | 3.05E-06 | 9.44E-05 |
| TPPP3 | 4.87E-01 | 4.50E-02 | 1.30E-02 | 3.90E-02 | 4.50E-02 |
